# PLS-R Calibration Models for Wine Spirit Volatile Phenols Prediction by Near-Infrared Spectroscopy

**DOI:** 10.3390/s22010286

**Published:** 2021-12-31

**Authors:** Ofélia Anjos, Ilda Caldeira, Tiago A. Fernandes, Soraia Inês Pedro, Cláudia Vitória, Sheila Oliveira-Alves, Sofia Catarino, Sara Canas

**Affiliations:** 1 Instituto Politécnico de Castelo Branco, Quinta da Senhora de Mércules, 6001-909 Castelo Branco, Portugal; 2 Centro de Estudos Florestais, Instituto Superior de Agronomia, Universidade de Lisboa, Tapada da Ajuda, 1349-017 Lisboa, Portugal; soraia_p1@hotmail.com; 3 Centro de Biotecnologia de Plantas da Beira Interior, 6001-909 Castelo Branco, Portugal; 4 Instituto Nacional de Investigação Agrária e Veterinária, Quinta de Almoínha, Pólo de Dois Portos, 2565-191 Dois Portos, Portugal; sheila.alves@iniav.pt (S.O.-A.); sara.canas@iniav.pt (S.C.); 5 MED—Mediterranean Institute for Agriculture, Environment and Development, Instituto de formação avançada, Universidade de Évora, Pólo da Mitra, Ap. 94, 7006-554 Évora, Portugal; 6 CQE—Centro de Química Estrutural, Associação do Instituto Superior Técnico para a Investigação e Desenvolvimento (IST-ID), Universidade de Lisboa, 1049-001 Lisboa, Portugal; tiago.a.fernandes@tecnico.ulisboa.pt; 7 DCeT—Departamento de Ciências e Tecnologia, Universidade Aberta, Rua da Escola Politécnica, 141-147, 1269-001 Lisboa, Portugal; 8 Faculdade de Ciências, Universidade da Beira Interior, 6201-556 Covilhã, Portugal; adriana-1998@hotmail.com; 9 LEAF—Linking Landscape, Environment, Agriculture and Food Research Center, Instituto Superior de Agronomia, Universidade de Lisboa, Tapada da Ajuda, 1349-017 Lisboa, Portugal; sofiacatarino@isa.ulisboa.pt; 10 CEFEMA—Center of Physics and Engineering of Advanced Materials, Instituto Superior Técnico, Universidade de Lisboa, Av. Rovisco Pais, 1, 1049-001 Lisboa, Portugal

**Keywords:** NIR, calibration models, PLS-R, volatile phenols, aged wine spirit

## Abstract

Near-infrared spectroscopic (NIR) technique was used, for the first time, to predict volatile phenols content, namely guaiacol, 4-methyl-guaiacol, eugenol, syringol, 4-methyl-syringol and 4-allyl-syringol, of aged wine spirits (AWS). This study aimed to develop calibration models for the volatile phenol’s quantification in AWS, by NIR, faster and without sample preparation. Partial least square regression (PLS-R) models were developed with NIR spectra in the near-IR region (12,500–4000 cm^−1^) and those obtained from GC-FID quantification after liquid-liquid extraction. In the PLS-R developed method, cross-validation with 50% of the samples along a validation test set with 50% of the remaining samples. The final calibration was performed with 100% of the data. PLS-R models with a good accuracy were obtained for guaiacol (r^2^ = 96.34; RPD = 5.23), 4-methyl-guaiacol (r^2^ = 96.1; RPD = 5.07), eugenol (r^2^ = 96.06; RPD = 5.04), syringol (r^2^ = 97.32; RPD = 6.11), 4-methyl-syringol (r^2^ = 95.79; RPD = 4.88) and 4-allyl-syringol (r^2^ = 95.97; RPD = 4.98). These results reveal that NIR is a valuable technique for the quality control of wine spirits and to predict the volatile phenols content, which contributes to the sensory quality of the spirit beverages.

## 1. Introduction

Volatile phenols are low molecular weight aromatic alcohols that comprise phenol and may include substituents such as alkyl, methoxyl, vinyl and allyl. These compounds can exist in foods due to a variety of mechanisms, as summarized by Schieber and Wust [1]. Some of these compounds are responsible for characteristic odor notes of various foods [1] and alcoholic beverages such as wine [2], whisky [3], rum [4] and aged wine spirit (AWS) [5]. Like other alcoholic beverages such as rum or whisky, Wine spirits are aged in wooden barrels, and the volatile phenols are among the most important compounds, in terms of sensory impact, extracted from the wood into the beverage. The main volatile phenols identified and quantified in AWS are guaiacol, eugenol, syringol, 4-methy-lsyringol, 4-allyl-syringol, 4-methyl-guaiacol and ethyl guaiacol, which are well related to odour notes such as smoky, clove, burnt, flowery and carnation, respectively [5]. Their amounts in the AWS are usually low (from traces to 1.5 g/L), increasing over time [6,7] and influenced by the wood species and toasting level, as well as the ageing system [7,8]. Although their low concentration in alcoholic beverages, these compounds have very low detection thresholds, and for this reason, several volatile phenols have been identified as critical odorants in wooden aged alcoholic beverages [3,4,5].

Gas chromatography (GC), coupled to an appropriate detection system (flame ionization detection (FID) or mass spectrometry (MS)), is typically used to analyse volatile phenols in alcoholic beverages. HPLC has also been used, although less extensively than GC [9]. These analyses are commonly preceded by an extraction step, which can be made through a variety of procedures such as liquid-liquid extraction [10], solid-phase extraction [11], solid-phase microextraction [12,13] stir bar sorptive extraction [14,15], dispersive liquid-liquid microextraction [16] and ultrasound-assisted emulsification-microextraction [17].

Near-infrared spectroscopy (NIR) is an analytical technique that uses the region of the electromagnetic spectrum between 12,500 and 4000 cm^−1^, and the collected spectrum of a sample comprises overtones and combination vibrations of molecules with different functional groups [18,19]. This analytical method has been applied in several matrices, namely foods and beverages. Compared to chemical analysis, NIR spectroscopy provides the ideal technology for quick and efficient analysis and has the advantage of being faster and requiring no sample preparation [20,21,22,23]. The most significant drawback is that the identification of small compounds is limited to a mass fraction more significant than roughly 0.1–0.5%. However, this also depends on the functional group(s) present in these compounds, which determines the magnitude of the absorption band shown in the NIR spectra. The intensity of a C–H vibration, for example, is substantially lower than that of an O–H vibration.

When paired with an appropriate chemometric methodology, NIR spectroscopy provides a rapid, non-destructive, and cost-effective method of food analysis that may be used for a wide range of products. It is used in the food sector to guarantee that the food being marketed meets the highest standards of food safety and hygiene and defend against false claims made by the food producer, processor, distributor, or retailer [20]. Its advantage is that NIR spectroscopy provides a spectrum that may be typical of a sample and may behave as a “fingerprint” by recording the response of specific chemical bonds (for example, O–H, N–H, C–H) to NIR radiation. Overtones of O-H or N-H stretching modes provide detailed data on intermolecular interactions, and NIR spectroscopy offers unique capabilities for analyzing hydrogen bonding. As a result, it is no surprise that NIR is commonly used to evaluate food compositional elements, but it can also be employed to determine more complicated attributes like texture and sensory characteristics [24].

PLS-R is a method for relating two data matrices to investigate complex problems and analyze available data more realistically. Many studies with different food products, was made using NIR data and PLS-R to perform calibration model [25,26], and in some cases with better responses than other regression techniques [27]. Additionally, the PLS-R technique is known to be affected by outliers in the data, and, in the present study, it is instrumental to eliminate possible outliers from the GC analyses. In the analyses of volatile compounds with low molecular weight, some outliers can occur and, with this technique, will be identified and eliminated more easily.

Concerning the alcoholic beverages, NIR analysis has been applied to assess the alcoholic strength of whiskies and vodkas [28] as well as other constituents of whiskies [28,29], rum and brandies [30], gin and vodka [31], and other distilled beverages [30,32,33,34] and to identify adulteration in distilled spirits [35]. Hanousek et al. [36] performed calibration models for major volatile compounds and phenols of wine spirits based on least squares regression. A recent study used NIR to distinguish wine spirits produced with two different wood species (oak and chestnut) and ageing technologies (barrel and alternative) with a precision of up to 90% [37].

**Figure 1:**
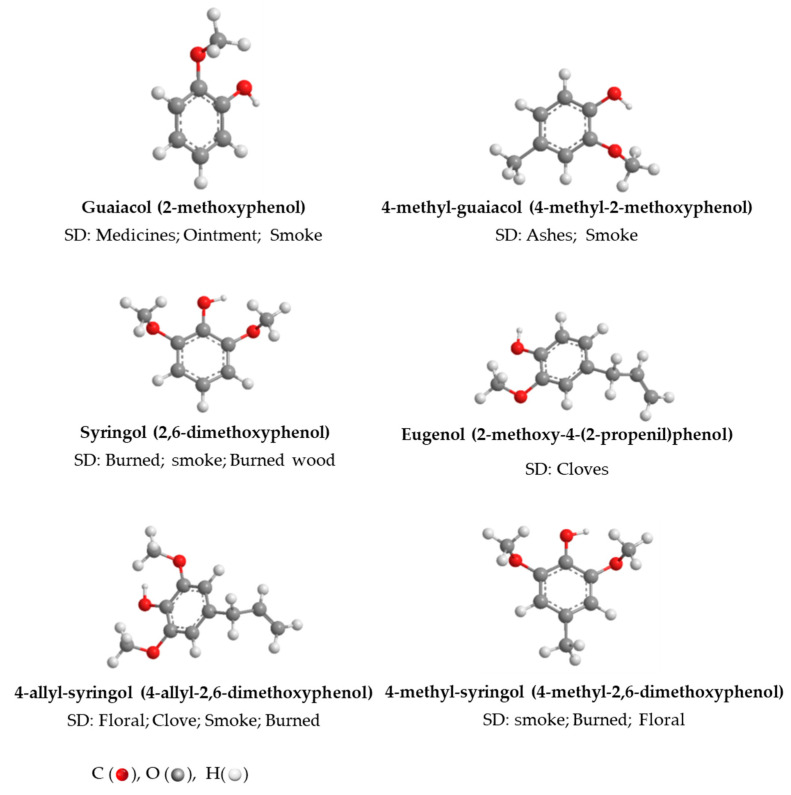
Chemical structure of volatile phenols studied in the AWS and their associated sensory descriptors (SD) [5].

Figure 1 shows the chemical structures and sensory properties of the most frequent volatile phenols in AWS, examined in this study.

This study aimed to assess the capability of NIR technology combined with chemometrics to perform calibration models to predict the content of volatile phenols in AWS.

## 2. Materials and Methods

### 2.1. Samples

The AWS samples used in this study were produced within the Oxyrebrand project-https://projects.iniav.pt/oxyrebrand (accessed on 14 December 2021) [6]. Briefly, samples resulting from ageing with different wood species (chestnut and oak), from traditional (250 L wooden barrel) and alternative technology (50 L glass demijohns with wood staves and micro-oxygenation-MOX) and two different periods of storage in the bottle were used. For the alternative systems, the 50 L demijohns with chestnut or oak wood staves underwent different micro-oxygenation conditions: flow rate of 2 mL/L/month during the first 15 days followed by 0.6 mL/L/month until 365 days; 2 mL/L/month during the first 30 days followed by 0.6 mL/L/month until 365 days; 2 mL/L/month during the first 60 days followed by 0.6 mL/L/month until 365 days; nitrogen application with a flow rate of 20 mL/L/month.

After the ageing process aforementioned, the AWS was bottled and stored for 2 months and analysed in the first stage of bottling (T0) and after 6 months (T6). For each modality, two essay replicates and three analytical measurements were used; a total of 120 samples were analysed, according to Table 1.

The use of these different AWS samples is intended to ensure a high variability to have accurate models that can be applied in a broader range of this kind of beverage.

### 2.2. Analytical Procedures

#### 2.2.1. Reagents

Anhydrous sodium sulfate and ethanol were acquired from Merck (Darmstadt, Germany), dichloromethane from Honeywell Riedel-de Haën (Steinheim, Germany), and silanized glass wool from Supelco (Steinheim, Germany).

The ultrapure water was achieved through the arium^®^comfort I equipment from Sartorius Lab Instruments, Goettingen, Germany.

GC-FID and GC-MS standards: guaiacol, eugenol, 3,4-dimethylphenol (internal standard), syringol, 5-methyl-2-hexanol (internal standard; IS) were bought from Fluka (Buchs, Switzerland); 4-methyl-syringol, 4-allyl-syringol were acquired from Aldrich (Steinheim, Germany); 4-methyl-guaiacol, were purchased from TCI (Zwijndrecht, Belgium).

#### 2.2.2. Quantification of Volatile Phenols in AWS

Prior to GC analysis, liquid-liquid extraction with ultrasonication was performed. The wine spirits samples (100 mL), previously diluted to 20% *v*/*v*, were added with internal standards and extracted with successive additions of 30, 10 and 10 cm^3^ dichloromethane and using ultrasonication according to the methodology described by Granja-Soares et al. [7]. The organic phases were collected, dried over sodium sulphate, filtered with glass wool silanized and then concentrated using a Büchi rotary concentrator (without vacuum at a temperature of 42 ± 0.5 °C) until a final volume of 0.25 mL. Each wine spirit sample was extracted in duplicate.

GC-FID analysed the concentrated extracts under the following chromatographic conditions: Agilent Technologies 6890 Series gas chromatograph (Wilmington, DE, USA) joined to a flame ionization detector (FID) and fitted out with a fused silica capillary column of polyethylene glycol (INNOWax of J&W Scientific, Folsom, CA, USA), 30 m, 0.32 mm i.d., 0.25 μm film thickness; split injection (1:25) of 0.8 μL of each extract; injector and detector temperatures (250 °C); carrier gas hydrogen (2.4 mL/min); oven temperature program: 3.5/min from 35 °C (6 min isothermal) to 55 °C, 7.5 °C/min to 130 °C, 5 °C/min to 210 °C (30 min isothermal). For each extract, three injections were done.

Hydroalcoholic solutions (20% *v*/*v*) of standards were extracted and analysed under similar conditions, and a calibration curve with five points was established for each compound. These curves were used for the quantification of volatile phenols in the AWS.

The compounds were identified by analyzing the extracts in GC-MS equipment (Magnum, Finnigan Mat, San Jose, CA, USA) under similar chromatographic conditions, with transfer line at 250 °C, working with electron impact mode at 70 eV and scanning the mass range of *m*/*z* 20–340. The compounds’ identities were determined by comparing the MS fragmentation pattern with reference compounds and with mass spectra in the NIST libraries.

### 2.3. Spectroscopic Measurements

The spectra of the AWS samples were obtained using a NIR spectrometer (MPA Bruker) in a transmitted light mode with 1 mm quartz cells. The samples were measured at 25 °C after 2 min in the instrument before scanning; the background was air-made. The samples were measured with an 8 cm^−^^1^ spectral resolution and 32 scans in the wavenumber range of 12,500 to 4000 cm^−^^1^ [32,37]. A background scan was performed after scanning a sequence of 10 samples.

### 2.4. Data Analysis

To ensure that the models were produced with a significant variability for the analytical determination, two principal component analysis (PCA) was performed: the first with the analytical determination identifying the different factor variance effects, and the second one with NIR spectra of AWS. The second PCA was also useful to identify the region that best discriminated the samples and, consequently, was the best to use in the models.

The model calibration analysis was performed with the average of two replicated spectra for each AWS sample.

The vector normalization pre-processes (SNV) were applied to all spectra used in the calibration models, which first normalizes a spectrum by calculating the average intensity value and then subtracting this value from the spectrum. Following that, new pre-treatments for model construction were tested. Briefly, multiplicative scatter correction (MSC); first derivative (1stDer); second derivative (2stDer), first derivative + multiplicative scatter correction (1stDer + MSC) and first derivative + straight line elimination (1stDer + SLS).

The cross-validation process was used in model validation with the general theoretical validation criterion leave-one-out method, which is more appropriate when a small dimension data set is used. The parameters used to identify a better calibration model were: r^2^—coefficient of determination (proportion of variance in the dependent variable that the independent one can explain); RPD—residual prediction deviation (by providing a metric of model validity, higher values correspond to better model’s predictive capacity); RMSEP—root means square error of validation; RMSECV—root means a square error of cross-validation, and RMSEC—root mean of the standard error of calibration.

Data pre-processing methods and selection of wavenumber ranges resulted in high predictability and precise estimation of volatile phenol in AWS.

The samples were divided into two sets, one for calibration (50% of data) and the other for validation (50% of data) after the model was tested with all values (100% of data), according to a similar methodology previously used [38].

The PCA for analytical data analysis was carried out using Statistica version 7.0 software (StatSoft Inc., Tulsa, OK, USA). Calibration models were made using OPUS 8.5.29 From Bruker Optik GmbH 2019. Spectral PCA analysis was performed using the UnscramblerX 10.5 (CAMO, Oslo, Norway).

## 3. Results

**Figure 2:**
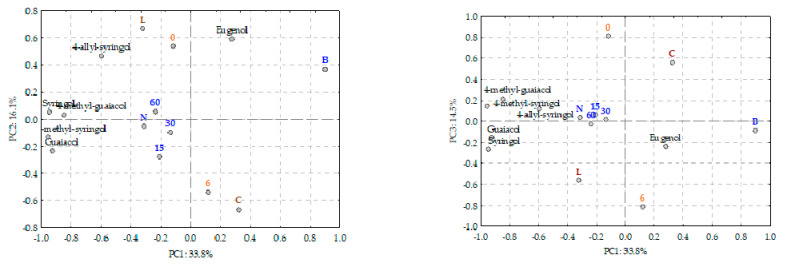
PCA representation of loadings and scores of all AWS samples and all volatile phenols analysed. Legend: C and L stand for the wood used in the ageing process, Chestnut and Limousin respectively; O15, 30 and 60 are the different micro-oxygenation modalities used in the alternative system; N—without micro-oxygenation; B—Barrel; 0—0 months in bottle; 6—6 months in bottle.

In this study, guaiacol, 4-methyl-guaiacol, eugenol, syringol, 4-methyl-syringol and 4-allyl-syringol contents in AWS presented a wide range of values (Figure 2) and significative variability given the different ageing modalities used as variability sources, which suggests a good data scattering.

**Figure 3:**
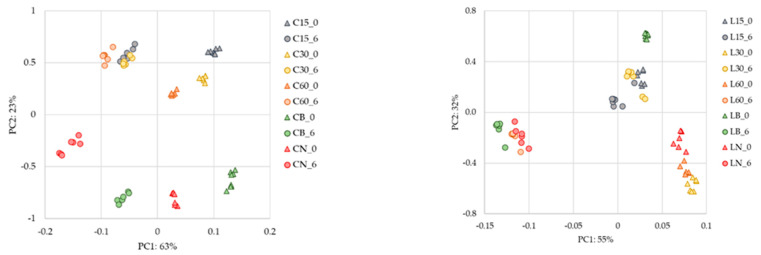
PCA was performed with spectral information of the AWS with chestnut (C) and with Limousin wood, acquired in NIR. Legend: C and L stand for the wood used in the ageing process, Chestnut and Limousin respectively; 15, 30 and 60 the different levels of micro-oxygenation used in the alternative system; N—no micro-oxygenation used in the alternative system; B—Barrel; 0—0 months in a bottle; 6—6 months in bottle.

Regarding Figure 3, it is possible to establish that the NIR spectra followed the trend of sample differentiation, which was also observed in Figure 2. However, NIR spectra showed that other compounds present in AWS could affect their relative position along the PCA axes [8,39,40]. In Figure 3, the AWS samples aged with chestnut wood and Limousin oak wood are presented separately to understand better.

**Figure 4:**
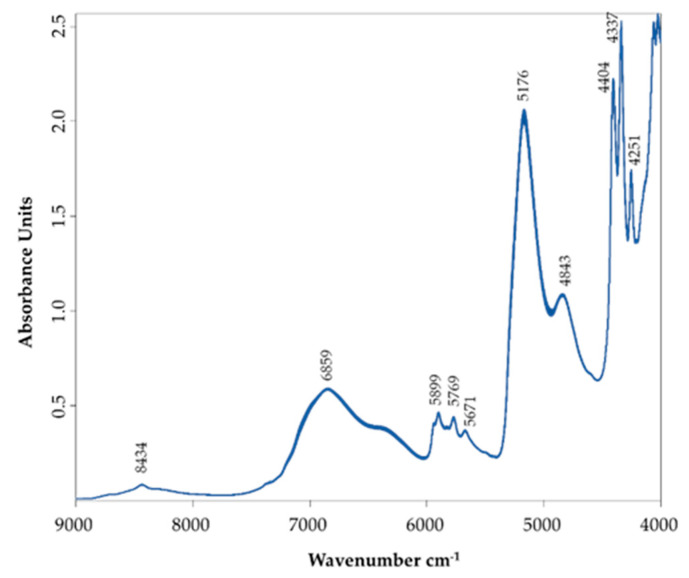
Representative absorption spectra of all AWS samples acquired in the NIR region measured against a background of air.

Figure 4 exhibits a representative NIR spectrum of the AWS, similar to those obtained by other authors for wine spirit, grape marc spirit, fruit spirits, whisky and vodka [28,29,37,40,41,42,43].

The water content in the spirits can be detected in the region around 6859 cm^−1^, which comprises the second overtones of the stretching νO–H band and a combination of deformation and stretching vibrations of the OH group (specifically water).

The peak with lower intensity near 8434 cm^−^^1^ is assigned to the second overtone of the C–H stretch of ethanol, one of the main compounds in AWS. This peak is also ascribed to the combination of the bending vibration of δO–H bend and the first overtone of the stretching νO–H has given the water influence [37].

The region from 5600 to 6000 cm^−1^ presents three small peaks ascribed to the νC–H stretch of the first overtones of CH_2_ and CH_3_ groups [22,43] and OH from aromatic groups [44].

At 6859 cm^−^^1^ occurs the second overtone of the stretching vibrations of ν(O–H) of water and ethanol as well.

The strong band at 5176 cm^−^^1^, characteristic of AWS [37], is related to a combination of stretching and deformation of the OH group and first overtones of water and ethanol and C–H stretch first overtones [43].

The peak at 4843 cm^−^^1^ can be assigned to aromatic C–H and –C=CH [44].

Volatile compounds extracted from the wooden barrel (mainly furanic and phenolic compounds) contribute to the flavour of the beverage [45,46]. Even in small amounts, soluble carbohydrates, most notably sugars, may contribute to the final flavour [46]. The ethanol, sugars and phenolic compounds have an absorption band at 4404 cm^−1^ related to the second overtone of stretching νC–H and νO-H overtones at 4338 cm^−^^1^ [47]. The bands at 4404 cm^−^^1^ and 4337 cm^−1^ are also related to the methanol content in the AWS [32,37]. The band at 4251 cm^−^^1^ is related to the combination of stretching and bending deformation of CH units of C–H(aromatic) and C–H(aryl) [48,49].

Table 2 presents the descriptive statistics (average, standard deviation, range, and coefficient of variation) for the volatile phenols, namely, guaiacol; 4-methyl-guaiacol, eugenol, syringol, 4-methyl-syringol, 4-allyl-syringol, content in the AWS samples used to develop the NIR calibrations. Table 3 shows the statistics of the prediction model for cross-validation of the calibration set and of the test set validation of the compounds above in the set of all samples analysed.

For the calibration models development, the entire infrared spectral region (12,000–4000 cm^−1^) was considered for spectral acquisition after eliminating the redundant spectra based on the spectral PCA analyses.

As shown in Table 2, a wide-ranging concentration value was found in the AWS for each volatile phenol, indicating a good scattering for such model development.

**Figure 5:**
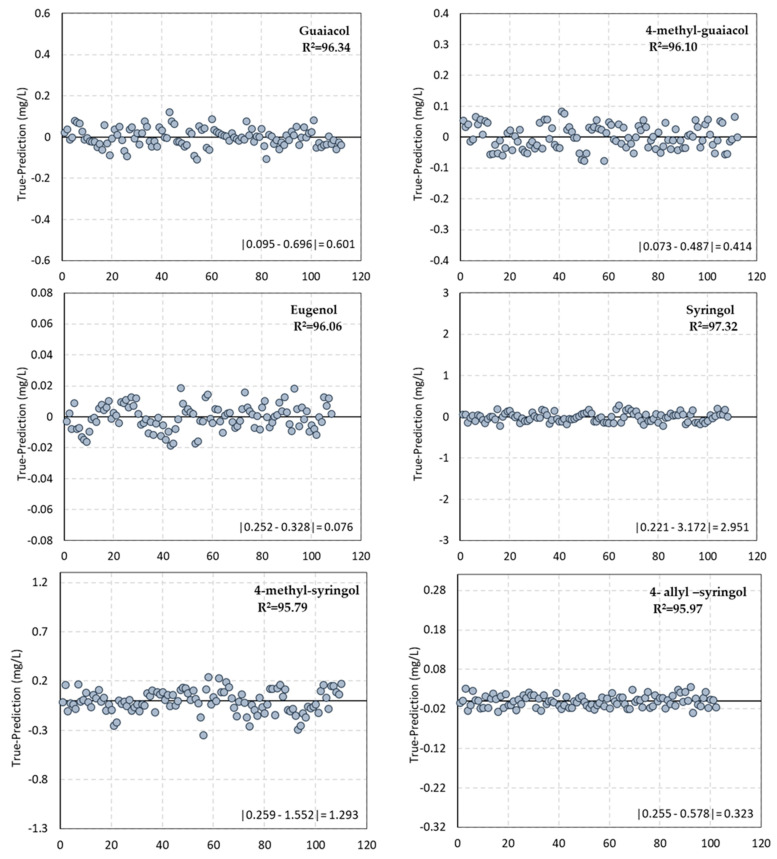
True value−Prediction value of each volatile compound analysed compared to the difference between the minimum and maximum a value.

The more accurate model, for each analysed compound, obtained with NIR raw spectral data regressed against their GC–FID determination is summarised in Table 3 for validation set (50% of the samples), cross-validation (50% of the samples) and calibration (100% of the samples). Figure 5 represents the deviation observed with the final calibration model.

The model selection was based on the analyses of all error parameters. Only the model with higher RPD, lower standard error of prediction of the test-set and calibration model (given by the root mean square error of cross-validation (RMSECV) and root mean square error of prediction (RMSEP)) and lower rank used in the prediction, were selected and presented. Bias analysis was also performed to confirm the adjustment of the model, and the value must be as nearer as possible to zero.

PLS was used to perform the calibration model with the more appropriate pre-treatments to increase the performance of the predictive models in the selected spectral range. Regarding Table 3, different spectral ranges were identified for each volatile phenol comprising wave number values from 9300 to 4500 cm^−1^.

Thus, for guaiacol quantification, the spectral range from 9118.1 to 5415.3 cm^−1^ was selected; for 4-methyl-guaiacol, three spectral ranges 8304.2 to 7347.7 cm^−1^ + 6869.4 to 5434.6 cm^−1^ + 4956.3 to 4478 cm^−1^, were selected; for eugenol, the spectral range was between 9337.9 and 5446.2 cm^−1^; for syringol, the spectral range between 6101.9 and 5446.2 cm^−1^ was selected; for 4-methyl-syringol, the spectral range was between 9160.5 and 4512.7 cm^−1^; for 4-allyl-syringol, the spectral range from 9353.3 to 7498.1 cm^−1^ + from 6101.9 to 5446.2 cm^−1^ were selected. Each chemical structure influences the analyte’s absorption bands’ position, shape, and size. Concerning the results mentioned above, the wavelength range selected in all calibration models was the region from 6000 to 5500 cm^−1^ ascribed to the νC–H stretch of the first overtones of CH_3_ and CH_2_ groups [42,49], and OH from aromatic groups [44]. According to the ageing time, these regions were also identified as good discriminants of wine spirits aged with different kinds of wood and ageing systems [37]. All these groups are presented in the volatile phenols studied, as shown in Figure 1, and some of them can even be differentiators when thoroughly examined. The hydroxyl groups arrangement (or even other substituent groups) at the aromatic phenolic skeleton has a significant impact on the absorption band shown in the NIR spectra, such as some of their chemical properties: dipole moment, bond dissociation enthalpy for the O–H bond, ionization potential or the antioxidant activity, among others. As a result, various skeleton and structural parameters, including the number and position of hydroxyl groups, the presence of other functional groups, their position in relation to hydroxyl groups, and stereochemical impediment, may affect the distinctive bands of each compound [50,51].

According to Jakubíková et al. [40], which used NIR spectroscopy to distinguish fruit spirits, the spectral region of 6050–5500 cm^−1^ is the more accurate to discriminate the different beverages analysed using PCA with linear discriminant analysis and general discriminant analysis models that giving 100% classification of spirits.

Concerning the pre-process selected, the one identified as better in the calibration model was the first derivative with 17 smoothing points combined with the multiplicative scatter correction or straight-line elimination (Table 3).

Regarding Table 3, all values of r^2^ are higher than 90.05%, which can be classified as excellent precision [52]. The values of r^2^ ranged between 90.05% for 4-allyl-syringol and 97.81 for syringol.

Several authors defined different threshold values for the accuracy of the model given by RPD that report the ratio between the standard deviation of the reference data of the validation set and the standard error of cross-validation prediction or the test set validation. According to Workman and Weyer [47], RPD must be higher than 2.5 to have good calibration. Conzen [53] states that a good calibration model must have an RPD higher than 3.0. In the present study, all models have values of RPD higher than 3.19.

The RPD values obtained for the analysed compounds ranged between 3.19 and 6.76 to predict 4-allyl-syringol and syringol, respectively. As far as we know, no studies were published about calibration models for volatile phenols. Therefore, it is only possible to compare with other volatile compounds of the AWS, but even these are scarce in the bibliography.

As aforementioned, the RMSEs (root mean square errors) of the validation set, cross-validation and calibration was also used to evaluate the ability of the PLS-R models developed to predict these parameters. All obtained values are low, denoting an accurate calibration model.

The NIR spectroscopy ability to monitor the distillation process of ethanol and methanol (two compounds that have legal limits for this beverage) from wine has been demonstrated by Dambergs et al. [54]. In this case, the more relevant regions studied for methanol and ethanol were 4401 cm^−1^ (related to CH combinations from the CH_3_ group) and 4337 cm^−1^ (associated with the CH_2_ group), respectively, which were also visible in the spectra obtained in the present study (Figure 4). At 5176 cm^−^^1^, the most significant peak is related to OH vibration combinations found in WS compounds and the volatile compounds that rise with the ageing process. These compounds are major volatiles of the WS, so they are easier to identify by NIR, and consequently, with more accurate models than those obtained for volatile phenols in this work. PLS and multiple linear regression (MLR) methods were tested for NIR calibrations using gas chromatography as the reference method in the study mentioned above. The PLS calibrations show better results with r^2^ of 0.96, a calibration error of 0.08% *v/v* for ethanol, and r^2^ of 0.99 and a calibration error of 0.06 g/L for methanol [54].

Yang et al. [32] proposed using two-dimensional NIR to determine the concentration of methanol in the white spirit combined with multivariate analysis, obtaining values of relative error of 2.97 and root mean square error of 0.079%.

In another research work [55], NIR was used to discriminate sugarcane spirits according to their origin using PLS-R, PLS combined with linear discriminant analysis, successive projection algorithm and genetic algorithm, which allowed identifying the authenticity of the studied beverages. Among the statistical approaches performed, the PLS-R model exhibited accurate values to predict the ethanol content of sugarcane spirits in the quality control process.

Figure 5 exhibits that the concentration value measured by GC (assumed as actual value) subtracted from the prediction value given by the corresponding proposed model for each volatile compound. Each graphic is represented in the spaces of the higher possible variance given by the minimum and maximum value difference observed in each analytical parameter. The results show the excellent performance of the models and the low deviation of the predicted value to the actual value one.

For the first time, this research shows the applicability of NIR spectroscopy to assess the volatile phenol’s contents, namely guaiacol, 4-methyl-guaiacol, eugenol, syringol 4-methyl-syringol and 4-allyl-syringol and confirms the ability of this technique to quantify those compounds in AWS.

## 4. Conclusions

The results attained in this study demonstrate that NIR spectroscopy can be used as an easy and quick method, without sample preparation and good reproducibility, to assess the content of volatile phenols in AWS. The performance of the models, given by the values of RPD, which are higher than 3.19 with a coefficient of determination higher than 90% and low root mean square error, are promising results for the use of this methodology at an industrial scale. However, further studies are needed to compare the ability of NIR with other methodologies, namely FTIR and RAMAN, using samples from other aged spirits, such as grape marc spirits, to increase the accuracy of the models and to extend this prediction analytical approach to other volatile compounds.

## Figures and Tables

**Table 1:** Sample characterization and number used in the model calibration.

		Chestnut Wood (C)		Oak Wood (L)		Total
		T0	T1	T0	T1	
(B) 250 L wooden barrel		6 *	6 *	6 *	6 *	24
50 L glass demijohns with wood staves with MOX	(15) with a flow rate of 2 mL/L/month during the first 15 days followed by 0.6 mL/L/month until 365 days	6 *	6 *	6 *	6 *	24
	(30) flow rate of 2 mL/L/month during the first 30 days followed by 0.6 mL/L/month until 365 days	6 *	6 *	6 *	6 *	24
	(60) a flow rate of 2 mL/L/month during the first 60 days followed by 0.6 mL/L/month until 365 days	6 *	6 *	6 *	6 *	24
(N) nitrogen application with a flow rate of 20 mL/L/month		6 *	6 *	6 *	6 *	24
Total		30	30	30	30	120

* Two replicates of each modality were carried and the analysis was made in triplicate (2 × 3 = 6).

**Table 2:** Statistics of the sample sets for guaiacol, 4-methyl guaiacol, eugenol, syringol, 4-methyl-siringol and 4-allyl-syringol quantification in AWS analysed.

Volatile Phenol	Number of Samples	N	Mean ± SD	Min–Max	CV (%)	LOQ ^1^
Guaiacol (mg/L)	Set1	56	0.491 ± 0.165	0.098–0.696	33.65	0.037
	Set2	56	0.489 ± 0.158	0.095–0.699	32.31	
	Set1 + Set2	112	0.487 ± 0.158	0.095–0.696	32.33	
4-methyl-guaiacol (mg/L)	Set1	56	0.279 ± 0.109	0.073–0.487	39.07	0.033
	Set2	56	0.280 ± 0.101	0.073–0.478	38.92	
	Set1 + Set2	112	0.279 ± 0.174	0.073–0.487	37.75	
Eugenol (mg/L)	Set1	54	0.291 ± 0.020	0.252–0.350	6.91	0.021
	Set2	54	0.290 ± 0.019	0.251–0.328	6.57	
	Set1 + Set2	108	0.289 ± 0.021	0.252–0.328	7.22	
Syringol (mg/L)	Set1	54	1.708 ± 0.705	0.221–3.172	41.31	0.029
	Set2	54	1.679 ± 0.683	0.244–3.106	40.66	
	Set1 + Set2	108	1.702 ± 0.695	0.221–3.172	39.65	
4-methyl-syringol (mg/L)	Set1	55	1.034 ± 0.383	0.274–1.552	37.04	0.034
	Set2	55	1.090 ± 0.395	0.259–1.536	36.21	
	Set1 + Set2	110	1.043 ± 0.393	0.259–1.552	37.66	
4-allyl-syringol (mg/L)	Set1	51	0.414 ± 0.076	0.273–0.55	18.32	0.043
	Set2	51	0.416 ± 0.078	0.255–0.578	18.80	
	Set1 + Set2	102	0.417 ± 0.075	0.255–0.578	17.87	

^1^ LOQ—limit of quantification; CV—coefficient of variation (CV = SD/mean); SD—standard deviation; min—minimum value observed in the corresponding set; max—maximum value observed in the corresponding set.

**Table 3:** Cross-validation and validation set results of the calculated models obtained for different determinations.

Volatile Phenol	Spectral Range (cm^−1^)	Pre-Process		Rk	r^2^	RMSEP	RMSECV	RMSEC	RPD	Bias
Guaiacol	9118.1–5415.3	1stDer + MSC	Set 1	10	96.80	0.0296			5.90	−0.0095
			Set 2	5	96.84		0.0270		5.63	0.0004
			Set 1 + 2	8	96.34			0.0298	5.23	
4-methyl-guaiacol	8304.2–7347.7 6869.4–5434.6 4956.3–4478	1stDer + SLS	Set 1	10	96.34	0.0233			5.36	−0.0052
			Set 2	10	92.70		0.0204		3.7	0.0006
			Set 1 + 2	10	96.10			0.0218	5.07	
Eugenol	9337.9–5446.2	1stDer + SLS	Set 1	7	95.30	0.0049			4.92	−0.0017
			Set 2	10	92.30		0.0053		3.59	0.0001
			Set 1 + 2	10	96.06			0.0044	5.04	
Syringol	6101.9–5446.2	1stDer + SLS	Set 1	9	97.81	0.1170			6.76	−0.0028
			Set 2	8	93.74		0.1560		4.50	−0.0028
			Set 1 + 2	10	97.32			0.1170	6.11	
4-methyl-syringol	9160.5–4512.7	1stDer + SLS	Set 1	10	94.88	0.0874			4.45	−0.0108
			Set 2	10	90.42		0.0653		3.23	−0.0024
			Set 1 + 2	10	95.79			0.0772	4.88	
4-allyl-syringol	9353.3–7498.1 6101.9–5446.2	1stDer + MSC	Set 1	8	90.05	0.0176			3.19	−0.0018
			Set 2	10	92.44		0.0243		3.64	−0.0011
			Set 1 + 2	10	95.97			0.0159	4.98	

MSC—multiplicative scatter correction; SLS—straight line elimination; 1stDer—first derivative; 2ndDer—second derivative; r^2^—coefficient of determination; RMSECV—root mean square error of cross-validation; RMSEP—root mean square error of prediction; RMSEC: root mean square error of calibration; RPD—ratios of performance to deviation; Bias—mean value of deviation, also called systematic error; Rk—rank.

## Data Availability

Not applicable.
